# SECP-Net: SE-Connection Pyramid Network for Segmentation of Organs at Risk with Nasopharyngeal Carcinoma

**DOI:** 10.3390/bioengineering10101119

**Published:** 2023-09-24

**Authors:** Zexi Huang, Xin Yang, Sijuan Huang, Lihua Guo

**Affiliations:** 1 School of Electronic and Information Engineering, South China University of Technology, Guangzhou 510641, China; 202221012396@mail.scut.edu.cn; 2 Department of Radiation, Sun Yat-sen University Cancer Center, State Key Laboratory of Oncology in South China, Collaborative Innovation Center for Cancer Medicine, Guangdong Key Laboratory of Nasopharyngeal Carcinoma Diagnosis and Therapy, Guangzhou 510060, China; yangxin@sysucc.org.cn (X.Y.); huangsj@sysucc.org.cn (S.H.)

**Keywords:** auto-context cascaded network, deep learning, segmentation of organs at risk of nasopharyngeal carcinoma, se-connection pyramid network

## Abstract

Nasopharyngeal carcinoma (NPC) is a kind of malignant tumor. The accurate and automatic segmentation of computed tomography (CT) images of organs at risk (OAR) is clinically significant. In recent years, deep learning models represented by U-Net have been widely applied in medical image segmentation tasks, which can help to reduce doctors’ workload. In the OAR segmentation of NPC, the sizes of the OAR are variable, and some of their volumes are small. Traditional deep neural networks underperform in segmentation due to the insufficient use of global and multi-size information. Therefore, a new SE-Connection Pyramid Network (SECP-Net) is proposed. For extracting global and multi-size information, the SECP-Net designs an SE-connection module and a pyramid structure for improving the segmentation performance, especially that of small organs. SECP-Net also uses an auto-context cascaded structure to further refine the segmentation results. Comparative experiments are conducted between SECP-Net and other recent methods on a private dataset with CT images of the head and neck and a public liver dataset. Five-fold cross-validation is used to evaluate the performance based on two metrics; i.e., Dice and Jaccard similarity. The experimental results show that SECP-Net can achieve SOTA performance in these two challenging tasks.

## 1. Introduction

Nasopharyngeal Carcinoma (NPC) is a kind of malignant tumor of high incidence in China [1], ranking first in attack rate beyond malignant tumors of the ear, nose, and throat. If the areas to be treated are not controlled precisely through radiotherapy (RT), normal organs may be affected, which has negative effects on the patients’ health. Computed Tomography (CT) images are a standard resource for the manual segmentation of OAR in the process of RT, which strictly limits the RT areas in the target region. Thus, RT will not damage normal organs. This shows that the segmentation of CT images plays an important role in clinical diagnosis and treatment. However, the workload of manual segmentation is incredibly heavy and time-consuming. If an automatic segmentation method for OAR can be designed for CT images, it will not only reduce doctors’ workload, but also improve the efficiency of clinical treatment.

To achieve automatic and accurate segmentation, methods of deep learning have been applied in recent years [2,3,4,5,6,7,8,9,10,11,12]. Various models based on convolutional neural networks (CNN) have been proposed for medical segmentation tasks and have achieved great success. Since it was first proposed, FCN [13] has become a basic frame of semantic segmentation. The encoder-decoder structure of FCN is capable of extracting image features from local information to higher spatial features through convolution and pooling measures. Based on FCN, U-Net [14], proposed in 2015, made some improvement on the skip-connection, which fuses features from the encoder with the corresponding parts from the decoder in the channel dimension to obtain more refined segmentation results. Thus, U-Net has been the most representative network of medical segmentation tasks in recent years. Many models based on U-Net have been proposed for obtaining more accurate results aiming at various application scenarios. Res-U-Net [15], inspired by residual connection, replaces the submodules in U-Net with residual modules to learn different features; Dense-U-Net [16] leverages the dense connection to maximally retain the information and gradient flow.

U-shape networks have intrinsic disadvantages, as follows: First, the skip-connection in U-Net is too simple because it directly introduces features captured by an encoder to the decoder instead of performing non-linear transformation; therefore, it will weaken its learning ability and result in classification errors due to noise. Second, U-Net does not extract or utilize sufficient multi-size information, and thus does not achieve a good performance for objects with a complex structure. Third, U-Net does not use global context information, and the extracted information will be diluted when transmitted to shallower layers.

To improve U-Net, many methods based on attention mechanisms have been proposed [17,18,19,20,21]. Attention U-Net [20] was a classical model based on U-Net which added a novel self-attention module to the skip-connection and adopted non-linear transformation to enhance the learning ability. Recently, many methods have further improved the accuracy of the segmentation by utilizing multi-scale information [22]. Among them, CE-Net [23] employed various blocks with different receptive fields to improve its ability in multi-size information extraction; UNet++ [24] proposed a kind of pyramid-like network to integrate the information from diverse levels, which made good use of global and multi-scale context information; CPF-Net [25] not only designed a global pyramid guidance module to combine multi-stage global context information, but also imported some convolution blocks with various dilation rates to capture the structure information.

To return to our specific segmentation task, there are multi-size OARs of NPC. Compared to big organs, the profile of small organs is not clear enough, due to their size; thus, it is more difficult to segment them using deep learning networks. In this paper, eight organs will be segmented; namely, eyes, temporal lobe, mandible, brain stem, parotid, submandibular, thyroid gland, and spinal cord. It is obvious that these organs have different shapes, and there is no doubt that we need a strong network to be able to capture the multi-size features. As previously mentioned, CPF-Net captures different sizes of features through several dilated convolution kernels of various dilation rates. In the original paper, the author chose three different kernels at most. However, in the segmentation task of OAR of NPC, there are eight organs of different shapes to be segmented. CPF-Net has insufficient parallel dilated convolutions for capturing multi-size information from all of the organs. It is not a suitable method for OAR segmentation tasks in NPC due to its intrinsic character.

Inspired by the above-discussion, a novel skip-connection module and a pyramid structure are proposed in this paper to overcome the three previously mentioned disadvantages. We named the proposed network SE-Connection Pyramid Network (SECP-Net). For the first disadvantage, we designed an SE-Connection (SEC) module for skip-connection. The SEC module makes use of the Squeeze-and-Excitation (SE) block [18] for its non-linearity and channel-wise attention to enhance the learnability and reduce useless noise influence from the input images, respectively. For the second and third disadvantages, we further propose a pyramid network that cooperates with the SEC module to merge information from multiple stages and to obtain the global context information.

The overall structure of this paper takes the form of five sections, including an introduction in Section 1. Section 2 gives the details of the proposed method. Section 3 shows the experiment details. Section 4 presents a discussion about the different methods. Finally, the conclusion is drawn in Section 5.

## 2. Method

**Figure 1:**
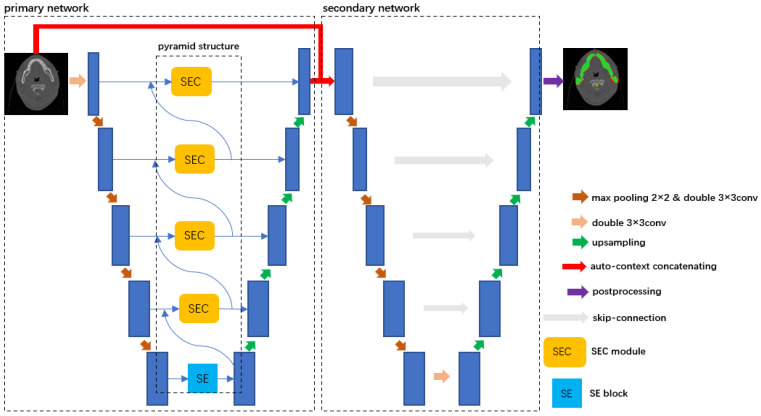
Overall structure of SECP-Net. After multiple convolutions and down-sampling, there are different sizes and levels of information captured in different stages for input images. We utilize SEC and pyramidal networks to fuse the information and extract more contributing features to segmentation by the channel attention mechanism of the SE block [18]. Then the information flow is transmitted to the decoder through a skip-connection. After the extraction of the primary part, we combine original inputs and the probability distribution from the primary network according to the auto-context, which is sent to the secondary U-Net for further segmentation and more accurate results.

Figure 1 shows the overall structure of SECP-Net, which is a cascaded network. The left part is the primary part of SECP-Net and the right part is the secondary network. The primary network, based on a U-shape network, consists of three parts: feature encoder, SEC module, and decoder. The SEC is located at the skip-connection between the feature encoder and decoder. In cooperation with the pyramid structure, the SEC can extract multi-size and global context information. The SEC also leverages the attention mechanism to highlight contributing features for segmentation. The secondary network is an original U-Net connected with the left part using the auto-context method, which increases the depth of the network to make the segmentation more accurate.

The encoder is used for capturing the feature information from input CT images while the decoder is used to restore images. In SECP-Net, there are two pairs of encoders and decoders in the primary network and secondary network, respectively. In the process of up-sampling, we conduct bilinear interpolation instead of deconvolution to avoid checkerboard artifacts [26].

**Figure 2:**
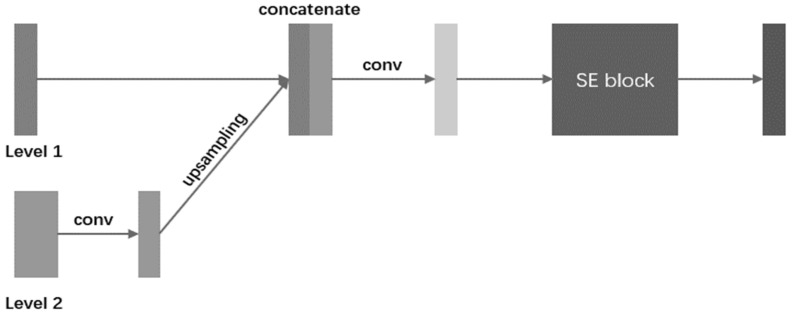
The illustration of SEC in a single stage. Multi-size information flow is fused stage by stage. The fusion information will be sent to the SE block for the acquisition of more contributing features. Then the output of the SE block is sent to the decoder by skip-connection.

For a U-shape network, its encoder can learn global context information by gradually increasing the respective field of convolution kernels, which contains a background of segmentation targets and their features. However, the information flow may be weakened when transmitted to shallower stages in the steps of up-sampling. U-Net utilizes the skip-connection to overcome this disadvantage. However, as mentioned previously in the first disadvantage of U-Net, the skip-connection of U-Net is so simple that the learning ability will be weakened due to non-linearity, and this type of structure will introduce noise, resulting in ambiguous errors for pixel classification. U-Net is not capable of extracting multi-size information, which causes the model to underperform in tasks of segmenting multiple targets according to the second and third disadvantages. In this paper, we present an SEC module and a pyramid network to address the above disadvantages. The structure of the SEC is shown in Figure 2.

In the SEC, the information between adjacent level stages is fused. As shown in Figure 2, Level 1 and Level 2 are feature maps captured in two continuous levels of network depth. Level 1 is from a shallower stage, and Level 2 is the output of the SEC from a deeper stage. Firstly, to keep the same channel space as Level 1, the feature maps of Level 2 will be convoluted (kernel size is 3 × 3 with the same padding and the stride is 1), and the feature maps after convolution will be up-sampled to the same size as Level 1. Secondly, we concatenate them together in a channel-wise way and utilize convolution (The kernel size is 3 × 3 with the same padding and the stride is 1) to obtain the fusion results. Finally, the fusion results are sent to an SE block, which is an attention mechanism that is proposed in [18]. SE has two steps; i.e., Squeeze and Excitation. To exploit contextual information, the squeeze operation extracts the global spatial information into a channel descriptor, which is achieved using global average pooling to generate channel-wise statistics. To make use of the information aggregated in the squeeze operation, the excitation operation employs a simple gating mechanism with a sigmoid activation to fully capture channel-wise dependencies. As SE can adaptively recalibrate channel-wise feature responses by explicitly modeling interdependencies between channels, our method uses an SE block after feature fusion to capture more contributing multi-size features with a channel-wise attention mechanism.

The process of extracting features of input stage by stage not only includes the acquisition of global context information, but of obtaining features of organs from small to large sizes. Therefore, we design a pyramid structure to make full use of the information. As shown in Figure 1, the dotted box is the proposed pyramid structure. The deepest part of the network has no information from a higher level. Thus, an SE block is added at the bottom of the pyramid structure to only extract features of big organs with a channel attention mechanism, and send them to the SEC to fuse them the with information from the corresponding encoder. Next, in this stage, we send the output of this SEC module to the decoder and shallower SEC module, which provides details for the up-sampling and fusing features of big organs from the deeper stage and smaller organs from the shallower stage, respectively. Similarly, multi-size and global context information is transmitted between neighboring layers. Thus, the information flow runs through the whole network, from bottom to top, which overcomes the second and third disadvantages. It provides an abundant contribution for image recovery and also results in more accurate segmentation.

In summary, the scientific contribution of the primary network is the SEC module and the pyramid structure. The SEC is an SE-Connection structure that is used for skip-connection. By embedding the SEC between encoders and decoders, the skip-connection has more non-linearity due to the more complex structure and learnable SE block. Therefore, the SEC can improve the network’s learning ability by increasing the non-linearity module. The pyramid structure can extract multi-size and global context information, and two kinds of useful information are transmitted between neighboring layers, and finally run through the whole network from bottom to top. Therefore, the cooperation between the SEC and pyramid structure can help to efficiently extract the multi-size and global context information.

There are eight organs of different shapes and sizes, which makes our segmentation of OAR of NPC difficult. If we use a pre-trained network to capture the ROI, we can focus on the areas of the targets to be segmented. Then, the output is sent to another deep network, resulting in a reduction in the learning difficulty of this secondary network. Thus, concatenating a pre-trained network and the other one makes it easier to segment the targets in ROI. Considering this, we design a cascaded network. As shown in Figure 1, we use a method called auto-context for connecting these two networks. The left dotted part of the SECP-Net in Figure 1 is the primary network that will be pre-trained. The right dotted part of the SECP-Net in Figure 1 is the secondary network. Auto-context [22] is also used for semantic segmentation. It combines the original input image data and the output probability distribution and then sends the fusion into the network. With this iteration, the final segmentation results will be more accurate.

## 3. Experiments

### 3.1. Dataset and Preprocessing

Two datasets are used to evaluate the system performance; i.e., a private dataset of the head and neck and a public LiTS dataset [27] of the liver.

The private NPC dataset used in this paper comprises CT images of the head and neck, which were collected by the Sun Yat-sen University Cancer Center (RDDA2020001435). It consists of over 40,000 CT images from 356 patients, and the resolution of a single image file is 512 × 512. We made masks containing 13 kinds of labels for the required segmentation targets, which include the left eye, right eye, left temporal lobe, right temporal lobe, left mandible, right mandible, brain stem, parotid left, parotid right, spinal cord, submandibular left, submandibular right, and thyroid gland. Table 1 shows the average volumes of these 13 organs. On this dataset, the eye, submandibular, and thyroid are rather small organs compared to others. We randomly divided the dataset into a training set and a test set: CT images from 285 patients are the training set and CT images from 71 patients are the test set. We applied five-fold cross-validation to the experiment, and the images were sampled into 256 × 256 while keeping the average aspect ratio due to insufficient GPU memory.

The liver dataset was provided by MICCAI 2017 LiTS Challenge and consists of 201 CT volumes, and 130 volumes were used for training. As the test set cannot be evaluated online on the official website of LiTS, we split the training set into two parts (100 patients for training and 30 patients for testing). The provided segmentation resulted in two different labels: liver and tumor. Like the multiple organs dataset, we applied five-fold cross-validation to the experiment, and the input size was resized into 256 × 256 to save the GPU memory.

### 3.2. Experimental Setting

In our experiment, we use multi-class cross-entropy loss as a loss function. The loss function L is described as:

(1) $$L=-\sum_{i}p_{i}\log_{2}(q_{i})$$

where i is the number of organs. The $p_{i}$ is the probability distribution of true labels and $q_{i}$ is the probability distribution of the results predicted by the deep networks.

To obtain the best performance of our model, we chose to utilize a strategy of learning rate decay, which is described as:

(2) $$lr={\left(\frac{1-epoch_{n}}{epoch_{total}}\right)}^{0.9}lr_{0}$$

where ${lr}_{0}$ represents the initial learning rate, which is 0.01, epoch n is the n-th training epoch, and ${epoch}_{total}$ represents the number of training epochs, which is 100. We chose SGD as the optimizer, in which the momentum and weight decay are set to 0.9 and 0.0001, respectively. These experimental settings are the same in CPF-Net [25]. In general, the larger the batch size, the better the performance. The maximum batch size is 16 because of the constriction of the computing device. The implementation of the proposed SECP-Net is based on the Pytorch platform and Nvidia GeForce RTX2080ti GPU with 12GB memory. We will release our codes on GitHub.

### 3.3. Evaluation Metrics

For the multiple organ datasets, we chose the Dice coefficient (Dice) for evaluating the performance of the models, which is the official evaluation standard for medical image segmentation. The higher the Dice, the more similar the results of the segmentation network and ground truth will be, and thus the better the performance. Dice can be described as follows:

(3) $$Dice=\frac{2\times |A\cap B|}{\left|A|+|B\right|}$$

where A and B are the results predicted by the deep networks and true results, respectively. ∩ represents the intersection. |·| is the number of pixels in the regions A and B.

For the LiTS challenge dataset, we used the official evaluation metrics: Dice per case and Dice global. Dice per case is used to accumulate the Dice of all the volumes (a set of CT slices for a single patient) and to average the sum. Dice global regards all volumes as a whole, and then it is calculated.

To evaluate the proposed framework’s performance, we also used two evaluation metrics used in this research field: precision and recall. They are calculated as follows:

(4) $$Precision=\frac{TP}{TP+FP}\times 100\%$$

(5) $$Recall=\frac{TP}{TP+FN}\times 100\%$$

where true positive (TP) indicates the number of positive classes correctly classified; false positive (FP) indicates the number of negative classes misclassified into positive classes; false negative (FN) indicates the number of positive classes misclassified into negative classes. As the background occupies the largest area, the accuracy and specificity will be dominated by the value of the background accuracy. Therefore, the accuracy and specificity are not used as evaluation metrics in our experiment.

We performed 5-fold cross-validation in both the ablation experiments and contrast experiments. The experiment was run 10 times, and the mean and standard deviation of metrics were calculated.

### 3.4. Training Scheme

To effectively train the cascaded network, SECP-Net, we trained our network according to the common method used for the cascade network. We first trained the primary part of the network, and then the secondary part. For the primary part, there were two steps: in step 1, we trained the backbone U-Net to convergence; in step 2, we added the SEC and pyramid structure, and then optimized the model with the initialized weights from step 1. Then, we fixed the parameters in the primary network and began to train the secondary part; thus, the whole network was fine-tuned.

Generally speaking, 2.5D or 3D deep networks perform better than 2D deep networks in medical image segmentation tasks. H-DenseUNet [28] was selected to test our dataset, which is a 2.5D model, and is considered as a SOTA method based on their published experimental results. As the GPU memory is 12Gbits, the batch size can only be 1 to avoid the overflow of memory in our experimental setting. This method resulted in a worse performance than the 2D deep network. The main reason is as follows: the lower the batch size is, the worse the performance achieved by the deep network. In addition, each slice is one training sample in a 2D network, but the total slice of one patient is one training sample in a 2.5D or 3D network. Therefore, the scale of the training set is bigger in the 2D deep network than in the 2.5D or 3D deep network. Based on these above reasons, we decided to use 2D networks in our experiments.

### 3.5. Results

We compared the proposed network with other remarkable models based on a U-shape network, including U-Net [14], Attention U-Net [20], CE-Net [23], UNet++ [24], CPF-Net [25], Res-U-Net [15], and Dense-U-Net [29]. Additionally, we also performed an ablation study to verify the validity of the SECP and auto-context. In the contrast and ablation experiments, U-Net is the baseline.

As is shown in Table 2, the values of the figures highlighted in black bold show the best performance corresponding to the organs to be segmented. They show that by improving certain shortcomings of U-Net, networks like Attention U-Net offer a better performance for every target organ than U-Net. It is obvious that UNet++ and SECP-Net perform better. This suggests that for the segmentation of multi-size organs, UNet++ and our method are more effective. Our SECP-Net has achieved the most excellent results in this experiment. Compared to the other methods, from U-Net to CPF-Net, in Table 2, SECP-Net achieves a significant improvement on small organs in terms of the Dice. For submandibular_L, the improvement of SECP-Net reached 5.06%, 2.57%, 7.92%, 1.72%, 4.57%, 3.56%, and 4.53%, respectively; for submandibular_R, the improvement reached 6.48%, 1.06%, 9.15%, 1.55%, 5.36%, 5.09%, and 6%, respectively; for thyroid, the improvement reached 5.04%, 2.82%, 5.93%, 2.24%, 3.83%, 5%, and 6.2%, respectively; for Eye L, the improvement reached 5.46%, 3.73%, 7.75%, 1.27%, 3.22%, 3.67%, and 4.05%, respectively; for Eye_R, the improvement reached 5.13%, 0.48%, 5.2%, 0.78%, 1.41%, 3.17%, and 2.53%, respectively. Small organs are difficult to segment using deep networks due to their size. In particular, when large organs are in a single CT slice together with small organs, traditional deep learning models tend to focus more on the big organs and ignore the small organs. By fusing and utilizing multi-size features from various organs, our method can balance the performance of organs of various sizes. The experimental results further prove that the proposed method successfully focuses on the information of small organs, and achieves a better performance in small organs. When segmenting the temporal lobe, mandible, and parotid left, UNet++ slightly outperforms SECP-Net. We investigated the significance of the differences in the average Dice obtained using our SECP-Net and seven medical image segmentation methods by applying a paired sample t-test with a 95% confidence level. The average improvement and significance are shown in Table 3. Our SECP-Net also achieved an improvement in the average Dice, and *p* < 0.5, which means that the statistical significance of the improvement is correct based on the statistical analysis. In our experiment, we only calculated the average precision and recall, as shown in Table 4. Our SECP-Net also achieved the best performance among all of the methods. Overall, in this experiment, SECP-Net showed the best performance, especially for small-sized organs. This is because our SECP-Net makes full use of the attention mechanism to focus on the most important information, and it refines the segmentation in the secondary network to obtain more accurate results.

As shown in Table 5 and Table 6, we evaluated different models on the LiTS dataset according to the dice per case and dice global. The values in black bold show the best segmentation performances for the liver and tumor. Obviously, our SECP-Net provided the best performance for both the liver and tumor. This also shows that by improving certain shortcomings of U-Net, other methods, like Attention U-Net, and UNet++, offer a better performance for liver and tumor than U-Net. There is no significant difference in the performance of the liver segmentation with dice per case among the various models. This is because the liver is large, so it is easy for deep networks to segment it. Table 5 shows that only the dice per case of the proposed SECP-Net is over 70%, whereas the others are all below 70%. SECP-Net performs far better than the other methods, achieving an average improvement of 9.5%, 7.26%, 5.96%, 4.19%, and 1.99% in dice per case, respectively. These big improvements show the effectiveness of our proposed SEC module and pyramid structure when it comes to multiple target segmentation, especially for a tumor that is much smaller than the liver. Our SECP-Net can capture and utilize information on multi-size organs effectively so that we can obtain more accurate results. The improvement in the dice global is far more than that of the dice per case as a result of its calculation method. Overall, SECP-Net also achieves the best performance in this dataset.

### 3.6. Ablation Study

The results of the ablation experiments are shown in Table 7. The baseline is the original U-Net. The values in black bold show the best segmentation performances.

(a) Baseline-concat: This represents the two original cascaded U-Nets. The left U-Net is used for the rough segmentation of ROI. The output of the primary U-Net is sent to the secondary network to refine the results.
(b) Baseline-auto-concat: We combine the classification probability from the primary network and original input images. Then, the combination is transmitted to the secondary network for more accurate segmentation, which achieves a better performance than a direct connection.
(c) Baseline-SEC: The SEC is embedded in an original U-Net. For small organs, such as the spinal cord, left submandibular, and right submandibular, this method performs far better than the baseline, which reaches 1.53%, 3.78%, and 5.6% for Dice, respectively. Comparing UNet++ with Baseline-SEC, Baseline-SEC outperforms UNet++, especially in small organs like the spinal cord, submandibular, and thyroid. For the spinal cord, the improvement reached 1.18%; for submandibular_L, the improvement reached 0.84%; for submandibular_R, the improvement reached 0.87%; for thyroid, the improvement reached 1.91%. This proves that the SEC is effective.
(d) Baseline-SEC-concat: Based on Baseline-SEC, we concatenate Baseline-SEC and an original U-Net. Baseline-SEC-concat achieves a better performance than Baseline-SEC. The network concatenation has a positive effect on the NPC segmentation task.

Finally, for the results of SECP-Net in the far-right column, the method proposed in this paper introduces auto-context for concatenated networks while tackling the disadvantages of U-Net, such as the lack of multi-size information and global context information. SECP-Net achieves the best performance in our ablation experiments.

### 3.7. Visualization Results

**Figure 3:**
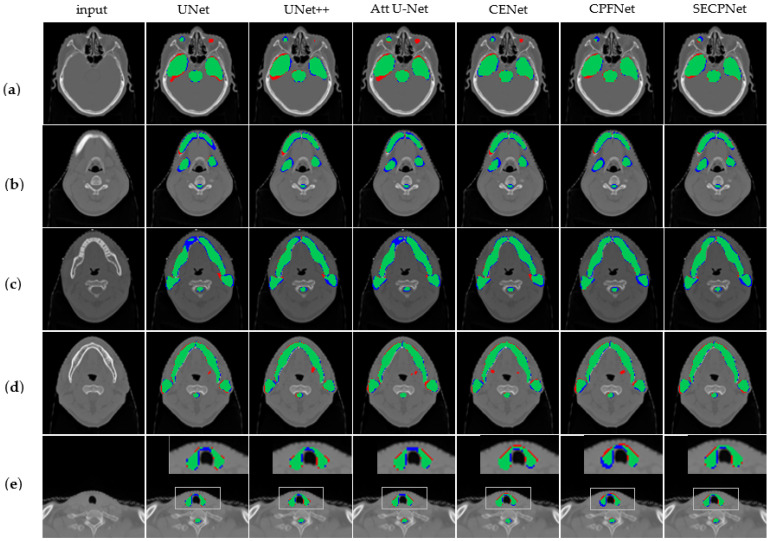
Visualization results of segmentation experiments. The blue part represents ground truth which is predicted as background (FN), the red part is the wrong results predicted by each model (FP) and the green part is an intersection of ground truth and the segmented results by the model (TP). From left to right: original image, U-Net, UNet++, Attention U-Net, CE-Net, CPF-Net, and our SECP-Net. (**a**) includes eyes, temporal lobe, and brain stem to be segmented from the top down; (**b**) includes mandible, submandibular and spinal cord to be segmented from the top down (submandibular and spinal cord are small organs); (**c**) includes mandible and parotid from top to bottom; (**d**) includes mandible and parotid from top to bottom; (**e**) includes thyroid and spinal cord to be segmented from top to down.

Figure 3 shows the visualization results of the different models on multiple organs.

As shown in Figure 3a, the eyes, temporal lobe, and brain stem are segmented. It is easily seen that U-Net, UNet++, Attention U-Net, and CE-Net all give incorrect segment results for the left eye, while the left eye does not exist in this single slice. Neither CPF-Net nor our SECP-Net makes such a mistake, while our SECP-Net provides a more accurate segmentation on the right eye and temporal lobe.

As shown in Figure 3b, the mandible, submandibular, and spinal cord are segmented, where the submandibular and spinal cord are small organs. Attention U-Net and U-Net both achieve poor results for the mandible. while UNet++, CE-Net, and CPF-Net all provide redundant segmentation parts of the mandible (the red part). Our SECP-Net gives a more accurate result, especially for the submandibular, due to it possessing more intersection parts.

As shown in Figure 3c, the mandible, parotid, and spinal cord are segmented. Compared to U-Net, Attention U-Net, and CE-Net, SECP-Net provides a more accurate segmentation for the left parotid due to the greater overlap part of the ground truth and the predicted.

As shown in Figure 3d, the mandible, parotid, and spinal cord are segmented. In fact, the submandibular does not exist in this image. However, nearly all of the methods provide an incorrect prediction regarding the submandibular (the red part in the middle position of images), with the exception of the proposed SECP-Net. SECP-Net performs far better than the above-mentioned models when it comes to small organs; namely, the submandibular.

As shown in Figure 3e, the thyroid and spinal cord are segmented, which are small organs. In comparison to UNet++ and SECP-Net, the other methods give rather less accurate results. The proposed SECP-Net makes full use of the multi-size feature information from big to small organs so that it can also obtain more accurate results for small targets.

## 4. Discussion

Attention U-Net [20] introduces the attention gate (AG) in the skip-connection. In the AG, the input features are scaled with the computed attention coefficients, and spatial regions are selected by analyzing both the activation and contextual information provided by the gating signal. It improves the skip-connection through the attention mechanism and can highlight the salient features useful for a specific task. However, Attention U-Net does not deal with global context information or multi-size information. Compared to Attention U-Net, our method not only uses the proposed SEC module and pyramid structure to extract the global multi-size information flow, but also utilizes the fusion structure and channel-wise attention mechanism in the SEC module to improve the skip-connection.

CE-Net [23] embeds the dense atrous convolution (DAC) block and the residual multi-kernel pooling (RMP) block in the deepest part of the network to overcome the disadvantage of lacking multi-size feature extraction. The DAC block has four cascade branches with a gradual increment of the number of atrous convolutions, from 1 to 1, 3, and 5; then, the receptive field of each branch will be 3, 7, 9, and 19. Therefore, the network can extract features from different scales. The proposed RMP block gathers context information with four different-size pooling kernels to overcome the disadvantage of the various sizes of objects in medical images [23]. However, CE-Net does not pay attention to the global context information. Compared to CE-Net, we consider both global context information and multi-size information in the proposed SECP-Net by adding the SEC module and designing a pyramid network. CE-Net does not improve the excessively simple skip-connection, while we set the SEC module in the skip-connection for more non-linearity and learnability.

UNet++ fulfills the blank part of the original U-Net using a dense connection from the low- to high-level stages in the network [24]. The receptive fields vary in different stages, which have varying sensitivity to diverse targets. In this case, UNet++ can capture features of different levels and overlie them in the channel dimension. In addition, global context information from deeper stages of the network can also be transmitted to shallower stages. Through iterative concatenation, this makes full use of the global context information. At the same time, this dense structure also makes the skip-connection more complex. Compared to UNet++, our SECP-Net designs a similar pyramid architecture for extracting multi-size information. In addition, the proposed SEC module improves the skip-connection through the channel-wise attention mechanism of SE, which makes the skip-connection more learnable. With the help of the attention mechanism, SECP-Net can emphasize more contributing features in the global multi-scale information flow, and ignore the useless information, while UNet++ just directly gathers global context information and multi-size features without any extra process.

In our OAR segmentation task of NPC, there are eight kinds of organs, of multiple shapes and sizes, to be segmented. CPF-Net proposes the global pyramid guidance (GPG) module, in which the global information flow is transmitted to the decoder by fusing the global context information from higher stages. In CPF-Net, multi-scale information is captured by a module named scale-aware pyramid fusion (SAPF), which consists of three parallel dilated convolution layers and is dynamically fused by two scale-aware modules [25]. CPF-Net primarily extracts multi-size organ information using several dilated convolution kernels with various dilation rates. When CPF-Net is applied to several kinds of organs, it performs excellently. On our multiple organs dataset, CPF-Net possessed inadequate dilated convolution kernels of various dilation rates for eight different kinds of organs. The proposed SECP-Net directly utilizes and fuses the multi-size information from different stages of the network, which can avoid the above disadvantage.

In summary, the main techniques and drawbacks of the five medical image segmentation methods are shown in Table 8. Attention U-Net, CE-Net, and UNet++ are all unable to completely overcome the disadvantages of U-Net. Although CPF-Net can improve all three of the shortcomings of U-Net, CPF-Net has the disadvantage of insufficient dilated convolution kernels to match the various organs in our segmentation task. SECP-Net meets the requirement of OAR segmentation of NPC. Furthermore, compared to the above methods, our auto-context concatenation improves the segmentation performance by introducing the probability distribution. As our SECP-Net is a cascaded network with two parts, which increases the depth of the network to make the segmentation more precise and accurate, our SECP-Net requires higher computation costs. However, the drawback of higher computation costs will be minimized with the development of computing devices.

## 5. Conclusions

As the traditional U-shape networks have an intuitive skip-connection, their performance is easily distorted by the presence of some noise due to the weakness of the learning ability. Moreover, U-shape networks cannot extract multi-size information and lack global context information. To overcome these disadvantages of U-shape networks, a novel pyramidal deep learning model, named SECP-Net, is proposed to automatically segment the OAR of NPC in CT images. In SECP-Net, the SE-Connection module and pyramid structure are used to capture the global multi-size information flow. The channel attention mechanism is fully utilized to highlight the contributing features, and the global context information is applied when concatenating networks with auto-context.

A private NPC dataset, which was provided by a local cancer center, was used to evaluate the performance of SECP-Net. Compared to the other competitive models—i.e., U-Net, Attention U-Net, UNet++, CE-Net, and CPF-Net—the experimental results show that the proposed SECP-Net can outperform the other competitive models. The same results can be achieved on the public LiTS dataset, which further confirms the effectiveness and generalization of our method. Moreover, the designed SEC module, pyramid structure, and auto-context concatenation were proven to be successful and effective parts for the OAR segmentation during the ablation study. With the exception of the channel attention, the local region attention requires further research, especially in establishing the relationship between the foreground and background. In the future, the image segmentation method based on U-shape networks will be combined with the transformer architecture to further improve the performance.

## Figures and Tables

**Table 1:** The average volumes of 13 kinds of organs.

Organ	Average Volume (cm^3^)	Organ	Average Volume (cm^3^)
left eye	8.4	parotid left	21.8
right eye	8.8	parotid right	22.4
left temporal lobe	134.3	spinal cord	23.1
right temporal lobe	125.8	submandibular left	9.3
left mandible	35.9	submandibular right	8.9
right mandible	37.2	thyroid gland	12.8
brain stem	30.8		

**Table 2:** The Dice Results of NPC Segmentation Task (%, Mean ± Standard Deviation).

	Methods	U-Net	Attention U-Net	CE-Net	UNet++	CPF-Net	Res-U-Net	Dense-U-Net	SECP-Net
Organs									
Temporal Lobe_L		86.55 ± 0.51	88.49 ± 0.60	86.01 ± 1.44	**89.04 ± 0.47**	88.17 ± 0.75	86.21 ± 0.45	86.63 ± 0.92	88.56 ± 0.66
Temporal Lobe_R		86.16 ± 0.61	86.66 ± 0.59	85.45 ± 1.20	**87.75 ± 0.50**	87.18 ± 0.81	87.33 ± 0.53	86.37 ± 1.18	87.55 ± 0.61
Eye_L		75.73 ± 0.73	77.46 ± 0.66	73.44 ± 1.11	79.92 ± 0.52	77.97 ± 0.63	77.52 ± 0.74	77.14 ± 0.46	**81.19 ± 0.77**
Eye_R		75.68 ± 0.82	80.33 ± 0.67	75.61 ± 1.08	80.03 ± 0.56	79.39 ± 0.67	77.64 ± 0.58	78.28 ± 0.79	**80.81 ± 0.71**
Mandible_L		86.17 ± 0.65	88.08 ± 0.53	84.82 ± 0.85	**88.38 ± 0.57**	87.70 ± 0.77	84.48 ± 0.82	85.37 ± 0.56	88.27 ± 0.58
Mandible_R		86.52 ± 0.67	87.10 ± 0.61	85.42 ± 0.79	**88.66 ± 0.49**	88.04 ± 0.73	85.19 ± 0.86	85.61 ± 0.59	88.60 ± 0.52
Brainstem		82.39 ± 0.68	84.08 ± 0.59	81.22 ± 0.73	84.38 ± 0.54	82.22 ± 0.56	80.40 ± 0.44	82.44 ± 0.64	**85.55 ± 0.41**
Parotid_L		78.40 ± 0.78	79.48 ± 0.44	76.17 ± 0.77	**80.87 ± 0.61**	79.61 ± 0.48	79.25 ± 0.35	79.62 ± 0.32	80.35 ± 0.53
Parotid_R		77.34 ± 0.74	78.89 ± 0.46	77.87 ± 0.84	80.53 ± 0.70	78.77 ± 0.56	77.28 ± 0.41	78.44 ± 0.38	**80.61 ± 0.48**
Spinal cord		88.06 ± 0.35	87.94 ± 0.41	86.41 ± 0.60	88.41 ± 0.38	87.19 ± 0.53	88.17 ± 0.35	88.52 ± 0.65	**89.77 ± 0.29**
Submandibular_L		72.32 ± 1.13	74.81 ± 0.65	69.46 ± 1.21	75.66 ± 0.87	72.81 ± 1.07	73.82 ± 0.71	72.85 ± 0.81	**77.38 ± 0.89**
Submandibular_R		72.71 ± 1.24	78.13 ± 0.67	70.04 ± 1.31	77.64 ± 0.89	73.83 ± 1.16	74.10 ± 0.37	73.19 ± 0.42	**79.19 ± 0.85**
Thyroid		69.77 ± 0.64	71.99 ± 0.71	68.88 ± 0.84	72.57 ± 0.59	70.98 ± 0.53	69.81 ± 0.84	68.61 ± 0.59	**74.81 ± 0.39**
Ave		79.83 ± 0.73	81.80 ± 0.58	78.52 ± 0.98	82.68 ± 0.59	81.19 ± 0.63	80.09 ± 0.57	80.23 ± 0.64	**83.28 ± 0.59**

**Table 3:** Average improvement and significance of our SECP-Net versus seven medical image segmentation methods.

	Vs U-Net	Vs Attention U-Net	Vs CE-Net	Vs UNet++	Vs Res-U-Net	Vs Dense-U-Net	Vs CPF-Net
Average improvement of SECP-Net	3.45%	1.48%	4.76%	0.6%	3.19%	3.05%	2.09%
The significance of improvement	*p* < 0.5	*p* < 0.5	*p* < 0.5	*p* < 0.5	*p* < 0.5	*p* < 0.5	*p* < 0.5

**Table 4:** Average precision and recall of NPC Segmentation Task.

	U-Net	Attention U-Net	CE-Net	UNet++	CPF-Net	Res-U-Net	Dense-U-Net	SECP-Net
Precision	0.876	0.900	0.889	0.901	0.896	0.898	0.892	0.908
Recall	0.847	0.899	0.878	0.892	0.890	0.893	0.883	0.902

**Table 5:** The Dice per case on LiTS (%, Mean ± Standard Deviation).

	Methods	U-Net	Attention U-Net	CE-Net	UNet++	CPF-Net	SECP-Net
Organs							
Liver		80.21 ± 1.38	81.59 ± 0.62	84.05 ± 1.26	84.73 ± 0.92	84.39 ± 0.83	**85.47 ± 0.69**
Liver Tumor		62.12 ± 1.55	64.36 ± 0.74	65.66 ± 1.37	67.43 ± 0.53	69.63 ± 0.79	**71.62 ± 0.78**

**Table 6:** The Dice global on LiTS (%, Mean ± Standard Deviation).

	Methods	U-Net	Attention U-Net	CE-Net	UNet++	CPF-Net	SECP-Net
Organs							
Liver		81.65 ± 1.23	82.73 ± 0.73	83.55 ± 1.34	85.43 ± 1.02	84.78 ± 0.76	**87.82 ± 0.58**
Liver Tumor		68.06 ± 1.61	72.27 ± 0.81	74.66 ± 1.42	75.59 ± 0.51	76.68 ± 0.83	**78.89 ± 0.69**

**Table 7:** The Dice of Ablation Experiments (%, Mean ± Standard Deviation).

	Methods	Baseline	Baseline-Concat	Baseline-Auto-Concat	Baseline-SEC	UNet++	Baseline-SEC-Concat	SECP-Net
Organs								
Temporal Lobe_L		86.55 ± 0.51	88.49 ± 0.66	88.54 ± 0.47	88.69 ± 0.41	**89.04 ± 0.47**	88.56 ± 0.58	88.56 ± 0.66
Temporal Lobe_R		86.16 ± 0.61	87.54 ± 0.64	87.52 ± 0.53	87.38 ± 0.46	87.75 ± 0.50	**87.78 ± 0.60**	87.55 ± 0.61
Eye_L		75.73 ± 0.73	79.78 ± 0.71	79.49 ± 0.74	79.52 ± 0.48	79.92 ± 0.52	81.06 ± 0.53	**81.19 ± 0.77**
Eye_R		75.68 ± 0.82	79.05 ± 0.73	79.21 ± 0.69	80.63 ± 0.47	80.03 ± 0.56	80.30 ± 0.49	**80.81 ± 0.71**
Mandible_L		86.17 ± 0.65	87.47 ± 0.42	87.54 ± 0.69	88.03 ± 0.73	88.38 ± 0.57	**88.56 ± 0.66**	88.27 ± 0.58
Mandible_R		86.52 ± 0.67	87.33 ± 0.56	88.10 ± 0.74	88.55 ± 0.76	**88.66 ± 0.49**	88.16 ± 0.68	88.60 ± 0.52
Brainstem		82.39 ± 0.68	84.06 ± 0.38	85.48 ± 0.70	84.72 ± 0.78	84.38 ± 0.54	85.18 ± 0.59	**85.55 ± 0.41**
Parotid_L		78.40 ± 0.78	80.33 ± 0.47	80.47 ± 0.67	80.04 ± 0.72	**80.87 ± 0.61**	80.10 ± 0.54	80.35 ± 0.53
Parotid_R		77.34 ± 0.74	79.16 ± 0.48	79.45 ± 0.61	80.31 ± 0.59	80.53 ± 0.70	80.20 ± 0.52	**80.61 ± 0.48**
Spinal cord		88.06 ± 0.35	88.42 ± 0.59	88.71 ± 0.51	89.59 ± 0.57	88.41 ± 0.38	89.67 ± 0.31	**89.77 ± 0.29**
Submandibular_L		72.32 ± 1.13	73.91 ± 0.67	75.44 ± 0.62	76.50 ± 0.78	75.66 ± 0.87	76.62 ± 0.91	**77.38 ± 0.89**
Submandibular_R		72.71 ± 1.24	74.71 ± 0.69	75.96 ± 0.53	78.51 ± 0.80	77.64 ± 0.89	78.21 ± 0.83	**79.19 ± 0.85**
Thyroid		69.77 ± 0.64	72.44 ± 0.49	71.84 ± 0.52	74.48 ± 0.60	72.57 ± 0.59	74.53 ± 0.43	**74.81 ± 0.39**
Ave		79.83 ± 0.73	81.75 ± 0.58	82.13 ± 0.62	83.09 ± 0.63	82.68 ± 0.59	83.10 ± 0.59	**83.28 ± 0.59**

**Table 8:** The main techniques and drawbacks of five medical image segmentation methods.

	Main Techniques	Drawbacks
Attention U-Net	The attention gate (AG) in skip-connection	It does not deal with global context information or multi-size information.
CE-Net	The dense atrous convolution block (DAC) and the residual multi-kernel pooling (RMP)	It does not pay attention to the global context information.
UNet++	The dense connection from low to high-level stages in the network	skip-connection is more complex
CPF-Net	The global pyramid guidance (GPG) module	It is difficult to design dilated convolution kernels of various dilation rates for different kinds of organs.
Our SECP-Net	The SE-connection module and the pyramid structure	It needs more computation costs

## Data Availability

The private NPC dataset is unavailable due to privacy or ethical restrictions.
